# Effects of Inflammation on Hippocampus and Substantia Nigra Responses to Novelty in Healthy Human Participants

**DOI:** 10.1038/npp.2014.222

**Published:** 2014-09-24

**Authors:** Neil A Harrison, Mara Cercignani, Valerie Voon, Hugo D Critchley

**Affiliations:** 1 Clinical Imaging Sciences Centre, Brighton and Sussex Medical School, University of Sussex Campus, Brighton, UK; 2 Sackler Centre for Consciousness Science, University of Sussex, Brighton, UK; 3 Department of Psychiatry, University of Cambridge, Cambridge, UK

## Abstract

Humans are naturally inquisitive. This tendency is adaptive, aiding identification of potentially valuable novel outcomes. The dopaminergic substantia nigra (SN) is implicated in the drive to explore novel stimuli and situations. However, infection and inflammation inhibit the motivation to seek out novelty. This likely serves to limit exposure to uncertain, potentially detrimental outcomes when metabolic resources are limited. Nevertheless, the neural mechanisms through which inflammation constrains novelty seeking are poorly understood. We therefore scanned 16 healthy participants (6 male, mean 27.2±7.3 years), using fMRI, once following experimental inflammation (intramuscular (i.m.) typhoid vaccination) and once after placebo (i.m. saline), with the aim of characterizing effects of inflammation on neural processing of novel and familiar place, and face stimuli. We specifically tested the effects of inflammation on the hypothesized roles of SN and hippocampus in novelty processing. Typhoid vaccination evoked a nearly threefold increase in circulating pro-inflammatory cytokine (interleukin-6) levels 3 h after injection, indicating induction of mild systemic inflammation. Enhanced hippocampal responses to novel (compared with familiar) stimuli were observed following both vaccine and placebo, consistent with intact central novelty detection. However, the normal bilateral reactivity of SN to stimulus novelty was significantly attenuated following inflammation. Correspondingly, inflammation also markedly impaired novelty-related functional coupling between the SN and hippocampus. These data extend previous findings of SN sensitivity to mild inflammation associated with changes in psychomotor responding, and suggest that inflammation-induced blunting of SN responses to hippocampal novelty signals may represent a plausible mechanism through which inflammation impairs motivational responses to novelty.

## INTRODUCTION

Humans are innately inquisitive and demonstrate a marked preference for novelty (Berlyne, 1955). This novelty preference is common to other species (Panksepp, 1998) and may motivate environmental exploration, enabling the identification of new sources of reward (Kakade and Dayan, 2002). However, novelty exploration is not without risk. Considerable energy can be consumed investigating ultimately unrewarding novel environments or foodstuffs, and exploration may increase exposure to predators or sources of infection (Lima and Dill, 1990). Interestingly, infection itself markedly reduces novelty exploration in rodents (Kusnecov *et al*, 1999). This reduction is a key component of sickness behaviors, a cluster of stereotyped behavioral adaptations that support, coordinate, and prioritize whole-organism immunological responses against the infecting organism (Hart, 1988). Pro-inflammatory cytokines including interleukin (IL)-1 can elicit sickness behaviors directly, impairing novelty preference, thereby mechanistically linking the body's inflammatory response to this biasing of behavior (Dunn *et al*, 1991). However, how this is mediated within the brain is currently poorly understood.

Inflammation attenuates novelty exploration across behavioral domains, including physical environments (Spadaro and Dunn, 1990), social interaction with juvenile conspecifics (Bluthe *et al*, 1992), novel objects (Haba *et al*, 2012) and preference for novel *vs* familiar foods (Pacheco-López and Bermúdez-Rattoni, 2011). This range of effects indicates an action on fundamental aspects of novelty processing. Human neuroimaging studies report increased hippocampal activity to novel stimuli (Stern *et al*, 1996), which is consistent with single-unit (Vinogradova, 2001) and c-Fos studies (Jenkins *et al*, 2004) in rodents, and also with theoretical models of the hippocampus as a comparator of incoming and previously experienced information (Knight, 1996, Lisman and Grace, 2005). Pro-inflammatory cytokines such as IL-1 can themselves impair hippocampal long-term potentiation (LTP; Katsuki *et al*, 1990) and synaptic strengthening (Bellinger *et al*, 1993), as well as spatial (Gibertini, 1996) and contextual (Barrientos *et al*, 2002) memory, suggesting a plausible locus for the effects of inflammation on novelty preference. Correspondingly, hippocampal stimulation can increase exploratory behavior (Flicker and Geyer, 1982). However, memory-enhancing effects of novelty appear to be mediated via coupling of hippocampus with brainstem dopaminergic nuclei (Lisman and Grace, 2005, Duzel *et al*, 2010). Novel stimuli increase burst firing in reward-sensitive mesolimbic dopaminergic cells (Horvitz *et al*, 1997; Schultz, 1998) and enhance activity within human substantia nigra (SN; Bunzeck and Duzel, 2006). Further, human SN responses to novelty correlate with a ‘novelty bonus' (Wittman *et al*, 2008) representing the intrinsic reward value of novel stimuli that is hypothesized to drive behavioral exploration (Kakade and Dayan, 2002). A role for dopamine in inflammation-induced attenuation of novelty preference is further suggested by the observation that IL-1-induced reduction in novelty seeking is blocked by the dopamine receptor antagonist sulpiride (Spadaro and Dunn, 1990).

Here, we use typhoid vaccination together with fMRI to dissect the neural basis to effects of inflammation on novelty processing. Typhoid vaccine is an established model of inflammation associated with a threefold increase in pro-inflammatory cytokines and induction of sickness behaviors 2–3 h after injection. It is a safe, licensed vaccine routinely administered to people traveling to parts of the world where the likelihood of acquiring typhoid fever is high. Specifically, we tested the prediction that inflammation impairs novelty preference via an action on ‘motivational' brainstem nuclei (SN) rich in dopaminergic neurons rather than via an action on hippocampal novelty detection processes.

## MATERIALS AND METHODS

### Participants

Seventeen healthy nonsmokers were recruited from University of Sussex campus advertisements. Volunteers were screened by a consultant psychiatrist (NAH) to ensure that they were healthy, had no previous history of any relevant physical or psychiatric illness, were taking no medication, had no recreational drug use in the previous 6 months and were nonsmokers. Volunteers who had received typhoid vaccine within 3 years or other vaccine within 6 months were excluded. One participant did not complete the second scanning session owing to technical difficulties. Of the remaining 16 participants (6 male, mean 27.2±7.3 years), 15 were Caucasian and 1 was Indian–Asian. All rated their general health as good, very good or excellent. Participants were advised not to consume caffeinated beverages or alcohol, avoid high-fat meals, and refrain from excessive exercise for 24 h before testing. They were asked not to take aspirin, ibuprofen or antibiotics for 14 days before testing. Written informed consent was obtained after complete description of the study to the participants. Study procedures were approved by the Brighton East National Research Ethics Committee.

### Study Design

We adopted a randomized, double-blind, crossover repeated measures design in which all participants underwent two separate sessions, an average of 7 days apart (as in previous reports, eg, Harrison *et al*, 2009). In the first session, participants were randomly assigned to one of two experimental conditions (typhoid vaccine or placebo saline injection). Eight participants received typhoid vaccination in the first session and eight participants received placebo injection. Baseline blood sample was taken and then injections of 0.025 mg *Salmonella typhi* capsular polysaccharide vaccine (Typhim Vi, Aventis Pasteur MSD, Berkshire, UK) or 0.5 ml of normal saline placebo administered intramuscular (i.m.) into the deltoid muscle. fMRI was performed 2–3 h after injection in a 60-min scanning session. During each session, participants performed two tasks. The present manuscript focuses on data acquired during an implicit novelty-processing task. Immediately after scanning, a second blood sample was taken (3 h after injection) for cytokine measurement. Body temperature was assessed at baseline and 3 h with an aural digital thermometer. The second fMRI scanning session was identical except that participants received the alternate injection (ie, typhoid vaccination if they previously received saline and vice versa).

### Experimental Model of Inflammation

We used the *S. typhi* vaccination model of inflammation that is previously known to induce a low-grade inflammatory cytokine response associated with a two- to threefold increase in peripheral IL-6 levels peaking between 2 and 3 h (Brydon *et al*, 2008). *S. typhi* vaccine 0.025 mg (Typhim Vi, Aventis Pasteur MSD) or 0.5 ml 0.9% sodium chloride placebo was administered i.m. into the deltoid muscle by a qualified doctor (NAH). There were no complications of either injection.

### Cytokine Analyses

Separate venepunctures were performed at baseline and 3 h after injection for both conditions. Blood (10 ml) was drawn into Vacutainer tubes (Becton Dickinson and Company, Franklin Lakes, New Jersey) containing ethylenediaminetetraacetic acid anticoagulant and centrifuged immediately at 1250 *g* for 10 min at room temperature. Plasma was removed, aliquoted, and frozen at −80 °C before analysis. Plasma IL-6, IL-1 receptor antagonist (IL-1ra), and tumor necrosis factor alpha (TNF*α*) were assessed using high-sensitivity ELISAs (R&D Systems, Abingdon, UK). The limit of detection of the IL-6 assay is 0.039 pg/ml, with intra- and interassay coefficients of variation (CVs) of 7.4% and 7.8%, respectively. The IL-1ra and TNF*α* assays had detection limits of 0.038 and 6.26 pg/ml with intra- and interassay CVs of 5.3% and 8.4%, and 7.8% and 5.3%, respectively. Cytokine analyses were performed using mixed measures ANOVAs in SPSS 20.0.

### Novelty Task

**Figure 1:**
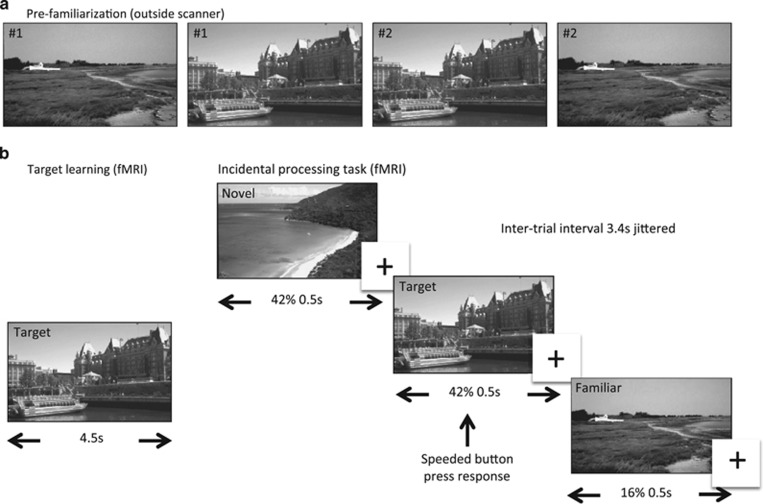
Task Structure. (a) Participants were pre-familiarized to 115 unique images (75 outdoor scenes as illustrated and 40 faces) before each of the two scanning sessions that were completed 2–3 h after blindly administered typhoid vaccination and saline (placebo) injection. (b) Each scanning session consisted of two separate counterbalanced scanning runs. On one run, participants were shown a single target scene (illustrated on left) followed by randomly presented familiar, target, or novel scenes. Forty-two percent of trials consisted of novel, 42% familiar, and 16% target images. Participants made a speeded button press to each presentation of the target stimulus. The second run was identical to the first except that participants were shown target, novel, and familiar face images. Stimuli were presented for 0.5 s with a jittered (mean 3.4 s) intertrial interval.

Immediately before each of the two fMRI scanning sessions, each participant was familiarized to 115 randomly selected gray-scale images (75 outdoor scenes and 40 faces). During this pre-familiarization phase, each image was shown twice for 3 s and participants were instructed to attend to each image carefully and told they would be asked about them later. Both scanning sessions consisted of two functional runs, in one run the participant was shown novel and familiarized scene images and in the other novel and familiarized face images. All stimuli were of neutral emotional valence. Each run began with presentation of a ‘target' outdoor scene or face stimulus that was shown for 4.5 s. Novel, pre-familiarized, and target stimuli were then presented in randomized order, and participants were instructed to respond with a speeded right-handed button press whenever the target image was presented (Figure 1). Targets accounted for 16% of all trials. Each picture was presented for 0.5 s with a jittered intertrial interval of 3400 ms. Different sets of images were used for each session counterbalanced across participants.

### Image Acquisition

Gradient-echo single-shot echo planar imaging was used to acquire T2*-weighted image volumes on a 1.5-T Siemens Avanto (Siemens AG Medical Solutions, Erlangen, Germany) scanner equipped with a 12-channel head coil. External restraint was used to minimize head movement. We acquired a total of 480 volumes each with 40 slices (interleaved ascending 2-mm slices with 1-mm interslice gap, echo time 50 ms: spatial resolution 3 mm × 3 mm × 3 mm). Slices were tilted −30° from the intercommissural plane to reduce orbitofrontal dropout owing to susceptibility artifact from the frontal sinuses (Deichmann *et al*, 2003). Magnetization transfer (MT) images were also acquired using a 3D gradient-echo sequence (matrix, 192, 192, and 64 slices; FoV, 200 × 200 × 160 mm; spatial resolution, 1.04 1.04 2.5 mm; TE=5 ms; TR=30 ms; flip angle=5°) to facilitate anatomical identification of the SN and to create an MT template. The MT template was derived by averaging the 16 individual MT images after they were spatially normalized to the standard MNI template supplied in SPM8. High-resolution inversion-recovery echo planar images were also obtained to aid image registration.

### Image Analysis

The fMRI data were analyzed with SPM8 (http://www.fil.ion.ucl.ac.uk/spm). The first 5 volumes were discarded to allow for T1 equilibration. Individual scans were realigned, unwarped, normalized, and spatially smoothed with an 8-mm full-width at half-maximum Gaussian kernel with standard SPM methods. High-pass frequency filter (cutoff 120 s) and corrections for auto-correlation between consecutive scans (auto-regressive 1) were applied to the time series. Each event was modeled by a standard synthetic hemodynamic response function at each voxel across the whole brain. Presentations of novel familiar and target images were modeled as separate regressors for both the face and outdoor scene image tasks.

First-level individualized design matrices were estimated in the following manner: effects of task (viewing novel or familiar face or external scene images) were computed on a voxel-wise basis for each participant for both vaccination and placebo conditions in the form of SPMs of discrete contrasts within the general linear model. Subsequent second-level analyses were performed on the SPM contrast images with a 2 (novel/familiar) × 2 (face/place) × 2 (inflammatory status) factorial ANOVA design to permit formal inferences about population effects.

### Regions of Interest

To address our prior hypotheses of selective effects of inflammation on hippocampal/parahippocampal or SN responses to stimulus novelty, we produced region of interest masks for both regions. The SN mask was custom-built based on the mean normalized MT image for all participants using MRIcron (http://www.mccauslandcenter.sc.edu/mricro/mricron) and the hippocampal/parahippocampal mask produced using the wfupickatlas (http://fmri.wfubmc.edu/software/PickAtlas).

### Multiple Comparisons

We used the cluster-extent threshold technique for reporting activated clusters outside of our predefined regions of interest (Slotnick *et al*, 2003). Specifically, we conducted a Monte–Carlo simulation using software written in MATLAB (The MathWorks, Natick, MA; https://www2.bc.edu/~slotnics/scripts.htm). After running 1000 simulations, we determined that for an individual voxel threshold of *p*<0.001, a cluster-extent threshold of 20 contiguous voxels was necessary to correct for multiple comparisons across the whole brain at a significance level of *p*<0.05. Thus, only clusters of activation meeting or exceeding a cluster-extent size of 20 contiguous voxels outside of our predefined regions of interest were considered significantly activated and reported.

### Psychophysiological Interaction SN Connectivity Analysis

To investigate effects of inflammation on changes in SN connectivity to novel stimuli, we first identified psychophysiological interactions (PPIs) of novelty reactivity and functional connectivity with the SN separately in placebo and inflammation conditions. Individual subject data was modeled using fixed-effects GLMs with three condition regressors (novel, familiar, and target conditions), one physiological regressor (first eigenvariate of all voxels within the bilateral SN mask to the contrast novel *vs* familiar), and a PPI regressor (constructed by multiplying this SN physiological regressor with the standard regressor of novel *vs* familiar). The resulting PPI regressors thus modeled differences in SN coupling across the brain as a function of novelty in placebo and inflammation conditions. Using this GLM, individual parameter estimate maps were generated for the contrast of interest: SN connectivity during novel compared with familiar stimuli in placebo and inflammation conditions. Parameter estimate maps for this contrast in both placebo and inflammation conditions were then compared in a second-level paired sample *t*-test.

## RESULTS

### Inflammatory Cytokine Responses to Vaccination

Across participants, typhoid vaccination evoked a robust inflammatory response with an ∼250% increase in plasma IL-6 from mean (±SE) 0.70±0.19 pmol/l at baseline to 1.74±0.24 pmol/l at 3 h (*t*_(15)_=5.20, *p*<0.001). The placebo condition evoked a much smaller rise in IL-6 from 0.67±0.16 pmol/l at baseline to 1.00±0.21 pmol/l at 3 h (*t*_(15)_=1.92, *p*=0.074), consistent with a physiological response to experimental stress (Brydon *et al*, 2004). This was confirmed by a significant treatment (inflammation *vs* placebo) by sample (baseline and 3 h) interaction for IL-6 (F_(1,15)_=12.44, *p*<0.003). Increases in plasma TNF-*α* or IL-1ra did not reach significance, consistent with previous findings (Brydon *et al*, 2008). No subject had previously received typhoid vaccination, therefore these findings reflect primary immune responses. There was no significant effect of vaccination on core body temperature of participants. Mean change in body temperature after vaccination −0.06 °C and placebo 0.12 °C, treatment by sample interaction F_(1,15)_=1.15, *p*=0.30.

### Responses to Stimulus Novelty

**Figure 2:**
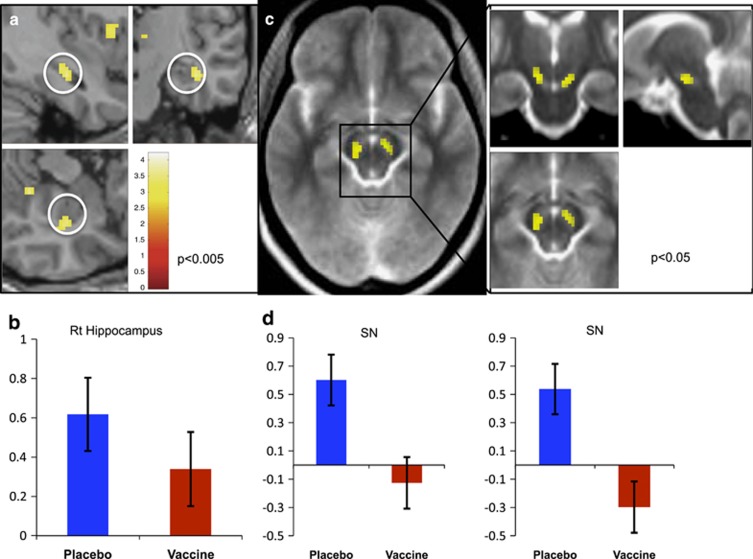
Hippocampal and substantia nigra (SN) responses to novel compared to familiar objects. (a) Right hippocampal region showing increased activation to novel compared with familiar objects (main effect of novelty) Family Wise Error (FWE) corrected *p*=0.025 for hippocampal region of interest. (b) Contrast estimates for the right hippocampal region following placebo (blue) and vaccine (red). (c) SN novelty × inflammation interaction, FWE corrected *p*=0.031 (right), *p*=0.051 (left). (d) Contrast estimates for the right and left SN following placebo (blue) and vaccine (red) demonstrating a significant reduction in SN responses to novel compared with familiar stimuli bilaterally following inflammation.

Perception of novel *vs* familiar images (main effect of novelty) was associated with significantly greater activity within the right hippocampal region of interest (small volume corrected (SVC) *p*<0.025; Table 1). This observation reinforces previously reported findings (Stern *et al*, 1996). It is noteworthy that hippocampal sensitivity to stimulus novelty was observed in both placebo and experimental inflammation conditions (Figure 2a and b). However, no significant main effect of novelty was observed within the SN region of interest. This result held even when a more permissive uncorrected threshold of *p*<0.05 was adopted, revealing only two voxels on the left side that did not survive SVC (*p*=0.308).

### Effects of Inflammation on Responses to Stimulus Novelty

To investigate whether inflammation selectively impaired hippocampal or SN responses to stimulus novelty, we next performed the critical inflammation × stimulus novelty interaction. Although the right hippocampus showed no change in sensitivity to novelty following inflammation, a discrete region within the right parahippocampus did show an attenuation of novelty responses following inflammation (Table 2). Interestingly, this region was adjacent to the area showing a main effect of novelty. Moreover, it was contiguous with a region we previously reported as showing reduced glucose metabolism following typhoid vaccine-induced inflammation (Harrison *et al*, 2014). Crucially, inflammation was associated with a marked reduction in bilateral SN responses to novel compared with familiar stimuli (SVC right *p*=0.031, left *p*=0.051; Table 2, and Figure 2c and d). Interindividual differences in induced IL-6 did not significantly correlate with this change, although did show weak trends (*p*=0.12 bilaterally) in the anticipated direction, that is, individuals with the greatest IL-6 response showed the greatest reduction in SN responses to novelty. Targets were correctly identified on 98.4% of trials, neither target identification nor response time (mean 538.0 ms) significantly differed between conditions, *p*>0.1.

### Functional Connectivity Analysis

To characterize these neurophysiological effects more closely, we finally tested the effects of inflammation on novelty-associated changes in SN connectivity. This analysis at first confirmed the novelty-associated increase in SN–hippocampus connectivity predicted by the Lisman and Grace, 2005 under basal (placebo) conditions (Table 3). However, this connectivity enhancement was not observed during inflammation. Indeed, paired sample *t*-tests demonstrated that inflammation significantly impaired the novelty-associated increase in SN–hippocampal connectivity observed under basal conditions (Table 3).

Together, our findings indicate that inflammation does not significantly impair hippocampal responses to stimulus novelty yet it does modulate the subsequent processing of this information by the SN and the functional integration of this information via the functional connectivity of these structures.

## DISCUSSION

Here we show that sub-pyrogenic inflammation selectively impairs human SN responsivity to novelty, without significantly affecting detection sensitivity to novelty within the hippocampus. Moreover, inflammation also blocked the increased functional connectivity between SN and hippocampus that occurs during novelty processing in noninflammatory states. Together, these data suggest that the impairing effects of inflammation on motivation and behavior (neophilia and novelty exploration) are likely mediated not through an action on hippocampal comparator processes involved in novelty detection, but through actions on the subsequent processing of this information within brainstem dopaminergic structures. Our findings provide empirical support for predictions arising from the novelty-related motivation of anticipation and exploration by dopamine (NOMAD) theory, which proposes that motivating effects of novelty are mediated via neuromodulatory dopaminergic pathways originating in brainstem (Duzel *et al*, 2010).

Converging evidence from humans, nonhuman primates, and rodents points to a specialized brain system for the detection of novelty, centered around the hippocampus and medial temporal lobe (MTL) memory system (Ranganath and Rainer, 2003). Both hippocampus and adjacent MTL structures respond robustly to novel stimuli (Stern *et al*, 1996; Bunzeck and Duzel, 2006; Howard *et al*, 2011). Hippocampal stimulation also increases exploratory behavior in a similar manner to novelty itself (Flicker and Geyer, 1982; Yang and Mogenson, 1987), an action that can be blocked by lesions to the ventral subiculum subregion of the hippocampus (Legault and Wise, 2001). However, human and animal studies also show that dopaminergic neuromodulation, originating from midbrain dopaminergic nuclei (SN and ventral tegmental area), enhance hippocampal synaptic plasticity in response to novel events, engendering a motivationally energizing effect on actions. Correspondingly, novel stimuli elicit increased activity not only within hippocampus but also SN and ventral striatum, regions implicated in generating motivational drive (Bunzeck and Duzel, 2006). A hippocampal–VTA/SN circuit is also proposed to control entry of information into long-term memory. In this model, sensory inputs enter hippocampus CA1 from entorhinal cortex. Information regarding detected novelty is outputted to both the midbrain (SN/VTA) and ventral striatum, where it is contextually integrated with other motivational goals. These trigger VTA projection neurons to release dopamine onto D1/D5 receptors at hippocampus CA1 synapses to enhance LTP and learning (Lisman and Grace, 2005). This specific translation of novelty detection into prospective effects on memory fits within a wider context of dopamine-mediated regulation of motivational behavior, including reward seeking (Pessiglione *et al*, 2006), addiction (Hyman *et al*, 2006), drive (Robbins and Everitt, 2007), and incentive motivation (Berridge, 2007). Moreover, the motivational effects of dopamine may manifest increased response vigor (Niv *et al*, 2007), which may in turn offset the increased risk associated with exploratory exposure. The ‘NOMAD' concept proposed by Duzel *et al*, 2010 attempts to unite these aspects of dopamine function in a model that informs our understanding of memory, ageing, and neurodegeneration. Correspondingly, our data provide further insight into the perturbation of adaptive responses to novelty by inflammatory mechanisms implicated in neuropathological processes, including progression of dementia (Perry *et al*, 2007) and depression.

Typically, inflammation is thought to impair memory through direct effects on hippocampal function (Yirmiya and Goshen, 2011). Our data now indicate that consequences of inflammation on the encoding and consolidation of new memories may also be mediated via indirect actions on the ascending arm of the hippocampal–VTA loop. Dopamine release within the hippocampus, from neurons originating in SN, enhances LTP and learning (Lisman and Grace, 2005). Our findings not only suggest that this interaction is compromised by inflammation but also can inform current controversies about how inflammation mediates memory impairment. Paradoxical effects of inflammation have been observed, for example, induction of low-grade inflammation (with lipopolysaccharide) in mice has been shown to enhance simple spatial discrimination learning on a T-maze side-discrimination task when one arm was always rewarded, yet impair it when the correct arm was rewarded on only 50% of trials (Sanderson *et al*, 2009). These observations require an alternative to a purely hippocampus-dependent account of inflammation-induced learning and memory deficits, and highlight the potential role of dopaminergic motivational mechanisms. Our data extend such observations to humans, presenting evidence for intact hippocampal novelty detection yet compromised dopaminergic signaling of stimulus novelty (plausibly reflecting motivational salience) during peripheral states of inflammation.

Interestingly, our findings of reduced SN novelty responses were observed in response to a relatively mild inflammatory challenge, suggesting particular sensitivity of novelty-processing mechanisms to inflammatory status. This finding accords well with recent rodent data demonstrating a marked reduction in responses to novel objects even after doses of intraperitoneal LPS that were insufficient to impair food intake, immobility on the forced swim test, or social interaction (Haba *et al*, 2012). Low-dose *Staphylococcus aureus* enterotoxin-B (a superantigen that induces T-cell activation and IL-2, interferon, and TNF release) also been shown to impair food intake in a novel but not familiar context without changing mobility or weight, suggesting a discrete effect on novelty responses unconfounded by illness anorexia (Kawashima and Kusnecov, 2002). Enhanced neophobia in this model was also reported towards nonedible inanimate objects reflecting an increase in neophobic behavior more generally (Kawashima and Kusnecov, 2002). Finally, temporal responses to LPS challenge have demonstrated impaired novelty responses for up to 24 h, long after changes in locomotor activity have returned to baseline, again supporting the suggestion that impaired novelty preference is one of the most vulnerable behaviors to peripheral inflammatory challenge (Haba *et al*, 2012). They also support the concept of a ‘behavioral immune system' designated not just to fight pathogens but also bias behavior to avoid the risk of infection (Schaller and Park, 2011).

Why peripheral inflammation should have such a profound effect on responses to stimulus novelty is currently unclear. However, may be usefully informed by consideration of the potentially adaptive advantages of sickness behaviors more generally. Immune responses demand substantial energy investment (Straub *et al*, 2010) and typically occur when food intake is reduced perhaps to restrict further exposure to a common source of infection or alternately restrict micronutrient availability required by some pathogens to replicate. It is perhaps unsurprising, therefore, that energy intense processes such as foraging and hunting, reproduction, and lactation are compromised during sickness responses to infection (Pacheco-López and Bermúdez-Rattoni, 2011). Increase in c-Fos expression in cortico–limbic structures such as the insula and amygdala following inflammatory challenge have been used to propose that immune-mediated enhancement of emotionality potentiates innate avoidant behavior reducing unnecessary danger when body energy resources are diverted (Pacheco-López and Bermúdez-Rattoni, 2011). However, long-lasting effects of immunoactivation on novelty responding have been demonstrated even in the absence of associated anxiety-like behaviors measured in open-field and elevated plus-maze tests, suggesting that they are unlikely to represent nonspecific anxiety responses (Haba *et al*, 2012). Our current data instead suggest a more discrete mechanism in which inflammation selectively impairs SN responses to novelty, a region rich in dopaminergic neurons that have been proposed to drive exploratory behavior with less beneficial effects on memory formation as a side effect.

We have also previously shown that change in response time on a Stroop task following typhoid vaccination correlated with increases in circulating IL-6 levels. Further, this correlated with reactivity of the SN (Brydon *et al*, 2008), suggesting a sensitivity of human SN to systemic inflammation. For humans, there is of course more to novelty than just foraging. Indeed, it has been argued that an interest in novelty and engagement of novelty-seeking behaviors ‘the lure of the unknown' is a driving force behind many of mankind's great discoveries (Knutson and Cooper, 2006). Our identification of a discrete action of inflammation on human SN responses to novelty offers a novel experimental model in which to further explore this.

## FUNDING AND DISCLOSURE

The authors declare no conflict of interest.

## Figures and Tables

**Table 1:** Main Effect of Viewing Novel *vs* Familiar Objects

**Side**	**Region**	**Coordinates**	***Z*-score**	***k***	***p*-Uncorr.**	**Corrected *p* (SVC)**
R	Hippocampus	[40 −16 −18]	3.50	26	<0.001	0.025
R	Parahippocampus	[22 −38 −14]	3.56	5	<0.001	0.074
R	Mid. frontal gy.	[36 46 36]	3.98	53	<0.001	NA
L	Inf. temporal gy.	[−42 2 −36]	3.87	34	<0.001	NA
L	Angular gy.	[−38 −82 30]	3.75	33	<0.001	NA
R	Ant. thalamus	[6 −10 4]	3.56	25	<0.001	NA

Abbreviations: L, left; NA, not applicable; R, right; SVC, small volume corrected.

**Table 2:** Interactions between Inflammation and Novelty Processing

**Side**	**Region**	**Coordinates**	***Z-*score**	***k***	***p-*uncor.**	**Corrected *p* (SVC)**
R	Substantia nigra	[8 −18 −14]	2.99	37	<0.05	0.031
L	Substantia nigra	[−10 −22 −14]	2.80	51	<0.05	0.051
R	Parahippocampus	[28 −38 −12]	3.74	14	<0.001	0.041
L	Hippocampus	[−20 −28 −12]	3.44	15	<0.001	0.096
R	Fusiform gy.	[36 −48 −22]	4.37	133	<0.001	NA
L	Mid. temporal gy.	[−52 0 −34]	4.07	33	<0.001	NA
R	Amygdala	[18 −2 −20]	3.74	123	<0.001	NA
L	Fusiform gy.	[−38 −34 −20]	3.86	50	<0.001	NA
R	Inf. occipital gy.	[46 −70 −10]	3.74	42	<0.001	NA
L	Post-central gy.	[−48 −38 60]	3.74	51	<0.001	NA
R	Amygdala	[18 −2 −20]	3.74	123	<0.001	NA
R	dorsal ACC	[8 32 52]	3.67	46	<0.001	NA
L	Sup. temporal sul.	[−52 −40 2]	3.65	29	<0.001	NA

**Table 3:** Effects of Novelty on SN Functional Connectivity

**Side**	**Region**	**Coordinates**	***Z-*score**	***k***	***p-*uncor.**	**Corrected *p* (SVC)**
*Placebo*						
L	Hippocampus	[−24 −24 −10]	3.99	24	<0.001	0.024
R	Hippocampus	[20 −12 −20]	3.94	59	<0.001	0.029
L	Retrosplenium	[−16 −50 8]	3.64	23	<0.001	NA
L	Retrosplenium	[−4 −58 10]	3.55	53	<0.001	NA
						
*Inflammation*						
R	Mid. temporal g.	[32 8 −34]	3.92	37	<0.001	NA
						
*Effects of inflammation (inflammation–placebo)*						
R	Hippocampus	[24 −22 −20]	4.17	23	0.001	0.014
L	Hippocampus	[−12 −34 −12]	3.63	26	0.001	0.184
